# Long‐lasting pathological consequences of overexpression‐induced α‐synuclein spreading in the rat brain

**DOI:** 10.1111/acel.12727

**Published:** 2018-01-30

**Authors:** Raffaella Rusconi, Ayse Ulusoy, Helia Aboutalebi, Donato A. Di Monte

**Affiliations:** ^1^ German Center for Neurodegenerative Diseases (DZNE) Bonn Germany

**Keywords:** axon, locus coeruleus, microglia, neurodegeneration, Parkinson's disease, stereology

## Abstract

Increased expression of α‐synuclein can initiate its long‐distance brain transfer, representing a potential mechanism for pathology spreading in age‐related synucleinopathies, such as Parkinson's disease. In this study, the effects of overexpression‐induced α‐synuclein transfer were assessed over a 1‐year period after injection of viral vectors carrying human α‐synuclein DNA into the rat vagus nerve. This treatment causes targeted overexpression within neurons in the dorsal medulla oblongata and subsequent diffusion of the exogenous protein toward more rostral brain regions. Protein advancement and accumulation in pontine, midbrain, and forebrain areas were contingent upon continuous overexpression, because death of transduced medullary neurons resulted in cessation of spreading. Lack of sustained spreading did not prevent the development of long‐lasting pathological changes. Particularly remarkable were findings in the locus coeruleus, a pontine nucleus with direct connections to the dorsal medulla oblongata and greatly affected by overexpression‐induced transfer in this model. Data revealed progressive degeneration of catecholaminergic neurons that proceeded long beyond the time of spreading cessation. Neuronal pathology in the locus coeruleus was accompanied by pronounced microglial activation and, at later times, astrocytosis. Interestingly, microglial activation was also featured in another region reached by α‐synuclein transfer, the central amygdala, even in the absence of frank neurodegeneration. Thus, overexpression‐induced spreading, even if temporary, causes long‐lasting pathological consequences in brain regions distant from the site of overexpression but anatomically connected to it. Neurodegeneration may be a consequence of severe protein burden, whereas even a milder α‐synuclein accumulation in tissues affected by protein transfer could induce sustained microglial activation.

## 1 INTRODUCTION

The protein α‐synuclein plays a key role in the pathogenesis of a number of age‐related neurodegenerative diseases, collectively referred to as synucleinopathies, that include Parkinson's disease, dementia with Lewy bodies, and multiple system atrophy. The initial link between α‐synuclein and human synucleinopathies was provided by genetic studies showing a causal relationship between a missense mutation, alanine to threonine, at position 53 (A53T) of the α‐synuclein gene (*SNCA*), and the development of autosomal‐dominant parkinsonism (Polymeropoulos et al., 1997). Further genetic studies identified additional *SNCA* missense mutations (A30P, E46K, H50Q, G51D, and A53E) linked to hereditary parkinsonism (reviewed in Petrucci, Ginevrino & Valente, 2016). They also revealed an intriguing association between familial parkinsonism and *SNCA* duplication and triplication (Chartier‐Harlin et al., 2004; Ibáñez et al., 2004; Singleton et al., 2003). In patients with these multiplication mutations, the severity of clinical presentation is correlated with gene dosage, because triplication carriers display more severe symptoms and earlier disease onset than patients with *SNCA* duplication (Ross et al., 2008). Pathological features triggered by *SNCA* multiplication are similar to those seen in idiopathic Parkinson's disease, including the degeneration of specific neuronal populations and the accumulation of α‐synuclein‐containing intraneuronal inclusions (Konno, Ross, Puschmann, Dickson & Wszolek, 2016). Taken together, these observations indicate that a sustained increase in protein expression is itself capable of triggering a gain of α‐synuclein toxic function, leading to development of Parkinson's disease‐like pathology. They also support the possibility that even transient and more localized elevations of brain levels of α‐synuclein could have deleterious effects and contribute to the development of synucleinopathies (Ulusoy & Di Monte, 2013).

Studies over the past few years have characterized an α‐synuclein property of likely relevance to its pathological role in human diseases: monomeric and aggregated α‐synuclein are able to pass across neurons from donor to recipient cells and can thus advance from brain region to brain region following anatomical connections (Desplats et al., 2009; Goedert, Spillantini, Del Tredici & Braak, 2013; Rey, Petit, Bousset, Melki & Brundin, 2013). In Parkinson's disease, this interneuronal protein transfer may account for the stereotypical pattern of progression of α‐synuclein pathology that, starting in the lower brainstem, affects higher and higher brain regions and ultimately reaches cortical areas (Braak et al., 2003; Goedert et al., 2013). Recent experimental work has demonstrated that brain spreading of α‐synuclein can be initiated by its overexpression. Caudo‐rostral protein transmission from the lower brainstem to the forebrain was prompted by targeted α‐synuclein overexpression within neurons of the rat or mouse medulla oblongata (Helwig et al., 2016; Ulusoy et al., 2013). Experimental evidence also revealed long‐distance α‐synuclein diffusion from the brain to peripheral tissues (i.e. the stomach wall) as a result of enhanced protein expression in the rat midbrain (Ulusoy et al., 2017). Thus, a pathogenetic mechanism by which increased protein load could promote the development and progression of a synucleinopathy may be via inter‐ and intraneuronal transmission of α‐synuclein and α‐synuclein species (e.g. oligomers) with toxic potential (Helwig et al., 2016; Kim et al., 2013; Rochenstain et al., 2014).

Age‐related synucleinopathies are characterized by a chronic disease course and progressive pathology, underscoring the importance of investigations into the long‐term consequences of neuron‐to‐neuron transmission and widespread diffusion of α‐synuclein. In this study, α‐synuclein spreading triggered by its overexpression in the rat medulla oblongata was assessed over a period of 1 year, significantly extending the observation time of earlier investigations, and was correlated with short‐ and long‐term markers of tissue injury. The new findings elucidate important factors that affect in vivo α‐synuclein transfer and its pathological outcomes under conditions of enhanced protein load. Data reveal for the first time that long‐term consequences of overexpression‐induced spreading include neurodegenerative changes and robust microglial and astrocytic reactions in tissues affected by “secondary” (i.e. post‐transfer) α‐synuclein burden.

## 2 RESULTS

### 2.1 Long‐term effects of α‐synuclein overexpression in the rat medulla oblongata

**Figure 1:**
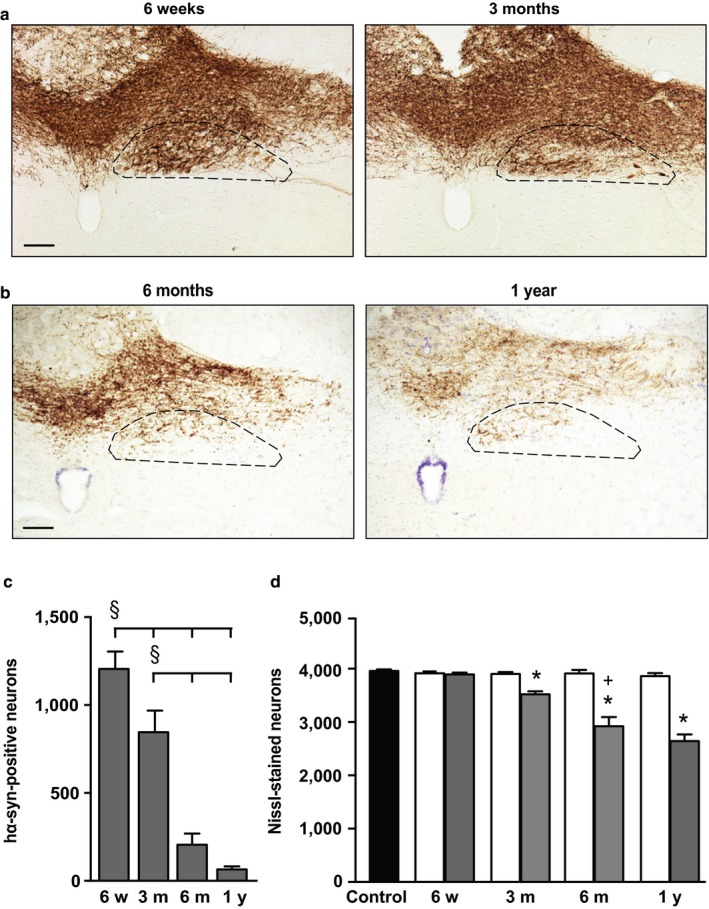
Time‐dependent degeneration of hα‐synuclein‐overexpressing neurons in the rat medulla oblongata. Rats received a single injection of hα‐synuclein‐carrying adeno‐associated viral vectors (AAVs) into the left vagus nerve and were killed at different time points after treatment. (a and b) Representative sections of the dorsal medulla oblongata from animals killed at early (a) or later (b) time points were labeled with an anti‐hα‐synuclein antibody. In each section, the left dorsal motor nucleus (DMnX) is delineated by dashed lines. Scale bar = 100 μm. (c) The number of hα‐synuclein‐positive neurons was counted in the left (AAV‐transduced) DMnX at different time points post‐treatment (*n* ≥ 5 animals/time point). Error bars indicate *SEM*. ^§^ *p *<* *.05 (one‐way ANOVA). (d) Stereological counts of Nissl‐stained neurons were carried out in the DMnX of untreated naïve rats (black bar; *n* = 4) as well as in the right (contralateral to the injection side, white bars; *n* ≥ 5) and left (ipsilateral to vagal injections, gray bars; *n* ≥ 6) DMnX of AAV‐treated animals. Error bars indicate *SEM*. *p <* *.05 vs. the contralateral side value at the corresponding time point (Student's *t* test). ^**+**^ *p *<* *.05 vs. the value at the earlier time point in the left DMnX (one‐way ANOVA)

Unilateral vagal injection of adeno‐associated viral vectors (AAVs) carrying human α‐synuclein (hα‐synuclein) DNA caused overexpression of the exogenous protein in the dorsal medulla oblongata (Figure 1a,b). More precisely, staining of medullary tissue with a monoclonal antibody specific for hα‐synuclein (syn211) showed immunoreactivity within cell bodies and axons of neurons in the dorsal motor nucleus of the vagus nerve (DMnX) ipsilateral (left) to the injection side; accumulation of hα‐synuclein was also detected within vagal afferent projections terminating into the nucleus of the tractus solitarius on the left and, to a lesser extent, the right medulla oblongata. While this distinct anatomical distribution of transduced neurons was observed at earlier (6 weeks and 3 months) and later (6 months and 1 year) time points after AAV administration, the intensity and extent of hα‐synuclein staining were time‐dependent. Robust and widespread labeling characterized samples at 6 weeks and 3 months (Figure 1a); in contrast, a weaker and less diffuse immunoreactivity was displayed by medullary sections at 6‐month and 1‐year postvagal injection (Figure 1b).

Changes in overexpression were also assessed by stereological counting of hα‐synuclein‐positive neurons in the left (AAV‐transduced) DMnX (Figure 1c). Counts revealed a significantly lower number of neurons (−30%) at 3 months as compared to 6 weeks post‐treatment. An even more pronounced loss occurred between 3 and 6 months and, by 1 year, only a few neurons immunoreactive for hα‐synuclein could be counted in the transduced tissue; indeed, values at 6 months and 1 year were 85% and 95% lower than the initial cell number at 6 weeks (Figure 1c). Reduced counts of hα‐synuclein‐positive cells were not due to a loss of transduction effect but rather to degeneration of neurons accumulating the exogenous protein. Data supporting this conclusion were obtained by counting the total number of Nissl‐stained neurons in the left DMnX. When compared to control values in naïve animals and in the right (contralateral to the injection side) DMnX, total neuronal counts were unchanged at 6 weeks but reduced by approximately 10, 25, and 30% at 3 months, 6 months, and 1 year, respectively (Figure 1d). Of note, at each time point, the decrease in Nissl‐stained neurons (e.g. −1,036 ± 172 cells at 6 months) matched the loss of hα‐synuclein‐immunoreactive cells (−999 ± 53 neurons at 6 months), underscoring a causal relationship between hα‐synuclein overexpression and neurodegeneration.

### 2.2 Overexpression‐induced spreading of α‐synuclein

**Figure 2:**
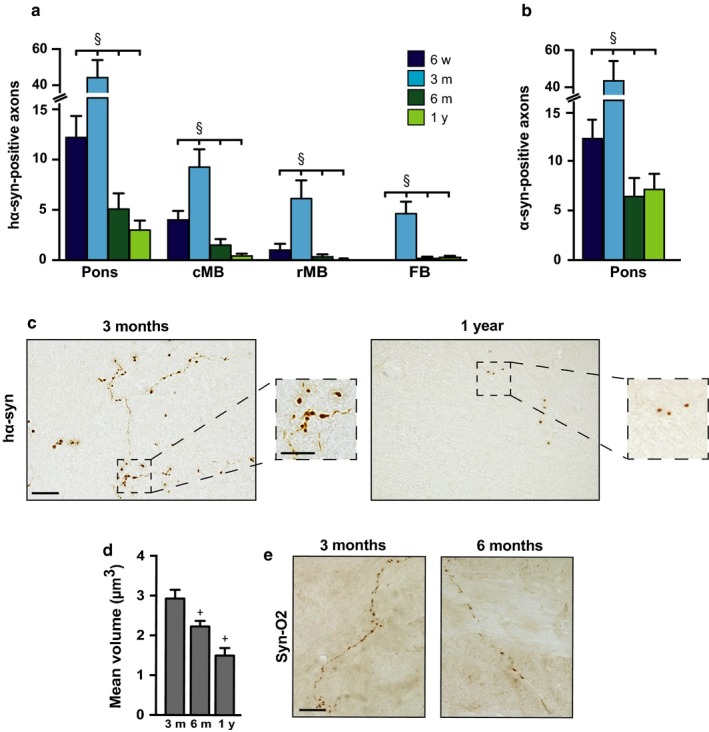
Long‐term reduction in hα‐synuclein‐loaded axons in brain regions affected by protein spreading. (a) The number of axons immunostained for hα‐synuclein with syn211 was counted in the brain of rats killed at different time points (*n* ≥ 4 animals/time point) after vagal AAV injections. Sections of the left (ipsilateral to the injection side) pons (Bregma: −9.6 mm), caudal midbrain (cMB; Bregma: −7.8), rostral midbrain (rMB; Bregma: −6.0), and forebrain (FB; Bregma: −2.4) were used for this analysis. Error bars indicate *SEM*. ^§^ *p *<* *.05 (one‐way ANOVA). (b) The number of axons immunostained for total (human plus rat) α‐synuclein with AB5038P was counted in sections of the left pons at different time points (*n* = 4 animals/time point) after vagal AAV injections. Error bars indicate *SEM*. ^§^ *p *<* *.05 (one‐way ANOVA). (c) Representative images show hα‐synuclein‐immunoreactive axons in the left (ipsilateral to the treatment side) pons of rats sacrificed at 3 months or 1 year after vagal AAV injections. The number of positive axons was markedly reduced at the later time point. The square boxes delineate two pontine areas shown at higher magnification in the smaller panels; at this higher magnification, the volume of axonal varicosities appears to be decreased at 1 year as compared to 3 months. Scale bar = 20 μm (large panels) and 10 μm (smaller panels). (d) The volume of axonal varicosities was measured in left pontine sections of rats killed at 3 months, 6 months, or 1 year after AAV injections (*n* ≥ 4 animals/time point). Data are expressed as geometric means ± 95% confidence interval. ^+^ *p *<* *.05 vs. the value at the earlier time point (Kruskal–Wallis test). (e) Representative images show pontine axons stained with the Syn‐O2 antibody, recognizing both oligomeric and fibrillar α‐synuclein. Sections of the left pons were collected from rats killed at 3 or 6 months after vagal AAV injections. Scale bar = 20 μm

Overexpression of hα‐synuclein triggers its neuron‐to‐neuron transmission from medullary donor neurons into recipient axons reaching the dorsal medulla oblongata from higher brain regions; through these axons, hα‐synuclein then spreads toward the pons, midbrain, and forebrain (Helwig et al., 2016; Ulusoy et al., 2013). The next set of analyses was designed to assess how death of donor neurons at later times post‐transduction affected hα‐synuclein brain propagation. Spreading was estimated by counting the number of axons immunoreactive for hα‐synuclein in brain sections at predetermined Bregma coordinates. A distinct pattern emerged, with progressive caudo‐rostral protein spreading occurring only during the first 3‐month post‐AAV injection (Figure 2a). At 3 months, hα‐synuclein had spread from the medulla oblongata first to the pons and caudal midbrain (by 6 weeks) and then to the rostral midbrain and forebrain. No further advancement occurred thereafter and, in fact, a pronounced decrease in counts of hα‐synuclein‐positive fibers was found in all brain regions at 6 months and 1 year; at these later time points, positive axons became sparse in the pons, rare in the caudal midbrain and virtually undetectable in the rostral midbrain and forebrain (Figure 2a). Axonal counts obtained in syn211‐stained sections were validated in a separate set of samples labeled with a polyclonal antibody recognizing both human and rodent α‐synuclein (AB5038P). The pattern and extent of protein spreading were similar between syn211‐ and AB5038P‐stained samples, and, in particular, data confirmed a dramatic loss of immunoreactive fibers between 3 and 6 months after AAV administration (Figure 2b).

### 2.3 Neuronal and glial pathology associated with α‐synuclein spreading

Spreading of hα‐synuclein and its consequent accumulation within recipient neurons were accompanied by morphological evidence of axonal pathology; syn211‐ or AB5038P‐labeled fibers appeared as tortuous threads with irregularly spaced and intensely labeled swellings (Figure 2c and Figure S1). The volume of these swellings is an indicator of hα‐synuclein burden (Ulusoy et al., 2013). Mean volumes of axonal varicosities were compared in pontine sections of AAV‐injected rats at different time points post‐treatment. Interestingly, they varied significantly, reaching their peak at 3 months and then declining by 25% at 6 months and 50% at 1 year (Figure 2c,d). Further characterization of axonal pathology was carried out using antibodies that recognize modified or aggregated forms of α‐synuclein. Phosphorylation at serine 129 is often used as a marker of α‐synuclein pathology (Neumann et al., 2002). However, when pontine, midbrain, and forebrain sections from AAV‐injected rats were stained for phospho‐Ser129 α‐synuclein, no immunoreactivity was detected at any time point post‐treatment (data not shown). To assess protein aggregation, tissues were labeled with two conformation‐specific antibodies: Syn‐O2 recognizes both early (oligomers) and late (fibrils) α‐synuclein aggregates, whereas Syn‐F1 specifically reacts against fibrillar α‐synuclein (Helwig et al., 2016; Vaikath et al., 2015). Immunoreactivity could be seen in sections labeled with Syn‐O2 but not Syn‐F1, indicating the presence of oligomeric but not fibrillar protein within abnormal axons (Figure 2e).

**Figure 3:**
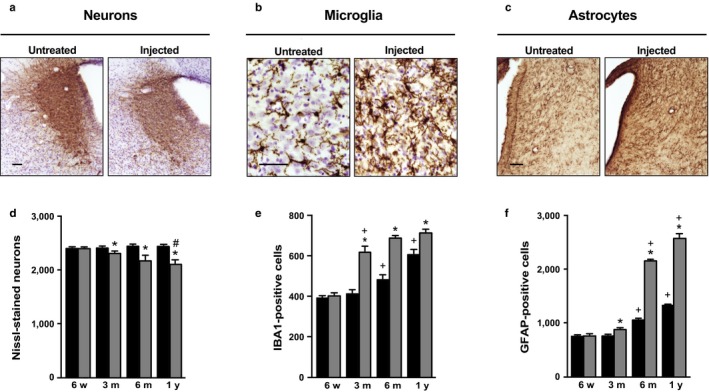
Neurodegeneration and gliosis as consequences of hα‐synuclein spreading in the locus coeruleus. (a‐c) Pontine sections were obtained from age‐matched naïve (untreated) rats and animals that were injected with AAVs in the left vagus nerve and killed at 6 months (microglia and astrocytes) or 1 year (neurons) post‐treatment. Sections were stained with antityrosine hydroxylase plus Nissl (a), anti‐IBA1 plus Nissl (b), or anti‐GFAP (c). Representative images show neuronal (a), microglial (b), and astrocytic (c) cells in the left locus coeruleus. Scale bars = 50 μm. (d‐f) Stereological counts of Nissl‐stained neurons (d), IBA1‐positive microglia (e) and GFAP‐immunoreactive astrocytes (f) were carried out in the locus coeruleus of untreated naïve rats (black bars; *n* ≥ 3/time point) and the left locus coeruleus of AAV‐injected animals (gray bars; *n* ≥ 3/time point). Error bars indicate *SEM*. *p <* *.05 vs. the value in age‐matched controls at the corresponding time point (Student's *t* test). ^**+**^ *p *<* *.05 vs. the value at the earlier time point in the respective treatment group (age‐matched controls or AAV‐injected rats) (one‐way ANOVA). ^**#**^ *p *<* *.05 vs. the value at the 3‐month time point (one‐way ANOVA)

Recipient axons accumulating hα‐synuclein under our experimental conditions would stem from brain nuclei that send direct projections into the dorsal medulla oblongata. The next set of analyses focused on a detailed assessment of pathological changes in two of these nuclei, namely the locus coeruleus in the pons and the amygdala in the medial temporal lobe (van der Kooy, Koda, McGinty, Gerfen & Bloom, 1984; Ter Horst, Toes & Van Willigen, 1991). The vast majority of neurons in the locus coeruleus is noradrenergic and displays immunoreactivity for tyrosine hydroxylase. Tyrosine hydroxylase labeling was less robust in the locus coeruleus of AAV‐injected rats killed at 1 year as compared to age‐matched naïve animals (Figure 3a). Following delineation of the locus coeruleus on pontine sections (Figure S2), the total number of neurons stained with cresyl violet was counted stereologically and compared in the left (ipsilateral to the injection side) locus coeruleus of rats killed at different times post‐AAV injection *vs*. the locus coeruleus of naïve age‐matched controls. While counts in animals killed at 6 weeks post‐treatment were similar to control values, a progressive neuronal loss occurred at later time points, with counts being reduced by 4% at 3 months, 11% at 6 months, and 15% at 1 year (Figure 3d).

**Figure 4:**
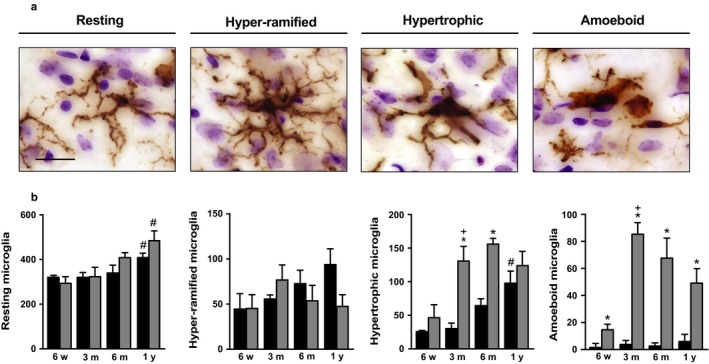
Changes in microglial phenotypes as a consequence of hα‐synuclein spreading in the locus coeruleus. (a) Pontine sections from AAV‐injected animals were immunolabeled with an anti‐IBA1 antibody. Representative images show typical morphological features of resting, hyper‐ramified, hypertrophic, and amoeboid microglia in the left locus coeruleus. Scale bar = 20 μm. (b) IBA1‐positive cells with morphological features of resting, hyper‐ramified, hypertrophic, or amoeboid microglia were stereologically counted in the locus coeruleus of untreated naïve rats (black bars; *n* ≥ 3/time point) and the left (ipsilateral to the AAV injection side) locus coeruleus of AAV‐injected animals (gray bars; *n* ≥ 3/time point). Error bars indicate *SEM*. *p <* *.05 vs the value in age‐matched controls at the corresponding time point (Student's *t* test). ^**+**^ *p *<* *.05 vs. the value at the earlier time point (one‐way ANOVA). ^**#**^ *p *<* *.05 vs. the value at the 3‐month time point in the respective treatment group (age‐matched controls or AAV‐injected rats) (one‐way ANOVA)

Next, the number and morphology of microglial cells were monitored in the left locus coeruleus of control (naïve) and treated rats. In control animals, counts of cells immunoreactive for ionized calcium‐binding adapter molecule 1 (IBA1, a marker of microglia) revealed age‐dependent changes because, as compared to values at 6 weeks and 3 months, the number of microglia was 25% and 50% greater at 6 months and 1 year, respectively (Figure 3e). In AAV‐injected rats, the number of IBA1‐positive cells was markedly elevated at 3 months and remained higher at 6 months and 1 year (Figure 3b,e). When compared to age‐matched control values, counts in these treated animals showed no change at 6 weeks, but significant increases at 3 months (+50%), 6 months (+40%), and 1 year (+15%). Subpopulations of IBA1‐labeled cells were also separately analyzed based on four distinct microglial phenotypes: (i) “resting”, surveying cells with small, round cell bodies and elongated, thin processes; (ii) “hyper‐ramified” cells with bigger, less circular cell bodies and dense ramifications of variable thickness; (iii) “hypertrophic” cells with big, irregular cell bodies and only a few, shorter, and poorly ramified processes; and (iv) “amoeboid” cells with an ovoid‐shaped body devoid of extensions or with one or two thick, unramified processes (Jimenez‐Ferrer, Jewett, Tontanahal, Romero‐Ramos & Swanberg, 2017; Torres‐Platas et al., 2014) (Figure 4a). The age‐dependent increase in microglial counts seen in the locus coeruleus of naïve rats was primarily due to an enhanced number of resting and hypertrophic cells (Figure 4b). Resting, hypertrophic but also amoeboid cells accounted for the higher microglial number in AAV‐injected animals (Figure 4b).

Astrogliosis is another typical reaction to brain tissue injury and was therefore evaluated as a potential pathological consequence of hα‐synuclein spreading and hα‐synuclein‐induced neurodegeneration. The number of astrocytes immunoreactive for glial fibrillary acidic protein (GFAP) was first estimated in the locus coeruleus of naïve rats. Similar to the effect of aging on microglia, astrocyte counts were found to be elevated by 40% and 75% at 6 months and 1 year, respectively, as compared to earlier time points (Figure 3f). AAV injections caused a small but statistically significant increase (+15%) in GFAP‐positive cells in the left locus coeruleus of rats killed at 3 months. This astrogliosis became much more pronounced, however, at 6 months and 1 year post‐treatment (Figure 3c); counts in animals killed at these later time points were approximately 100% higher than the corresponding values in age‐matched controls (Figure 3f). Of note, immunohistochemical analyses of either microglial or astrocytic cells in the locus coeruleus of AAV‐injected animals showed no evidence of accumulation of the exogenous hα‐synuclein protein within these cells (data not shown).

**Figure 5:**
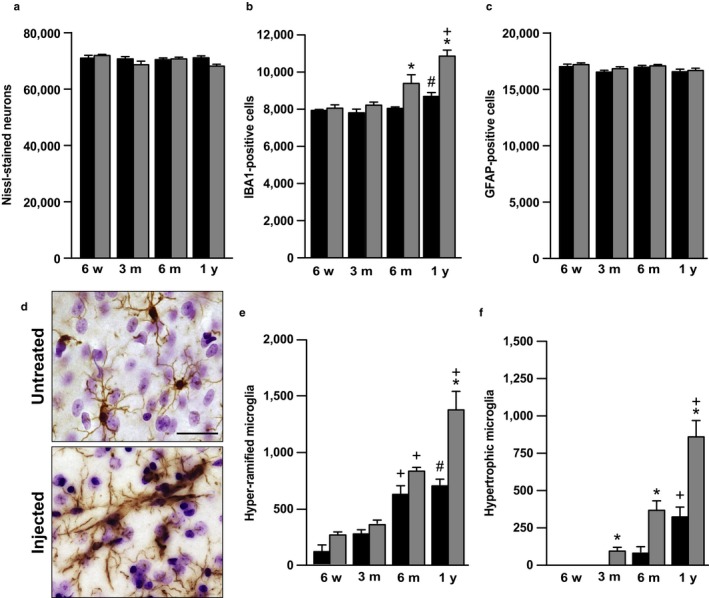
Microgliosis as a long‐term consequence of hα‐synuclein spreading in the central amygdala. (a‐c) Stereological analyses were performed on tissue sections encompassing the entire left central amygdala of rats that received a unilateral (left) vagal injection of hα‐synuclein‐carrying AAVs (gray bars). Animals were sacrificed at different time points post‐treatment (*n* ≥ 3 animals/time point). Comparisons were made with control values obtained from age‐matched naïve animals (black bars; *n* ≥ 3 rats/time point). The number of Nissl‐stained neurons (a), IBA1‐positive microglia (b), and GFAP‐immunoreactive astrocytes (c) was estimated. (d) Representative sections of the left central amygdala of an age‐matched naïve (untreated) rat and an AAV‐injected animal killed at 1 year post‐treatment were immunostained with an anti‐IBA1 antibody. Scale bar = 25 μm. (e and f) The number of IBA1‐immunoreactive cells with morphological features of hyper‐ramified (e) or hypertrophic (f) microglia was counted stereologically in the left central amygdala of age‐matched naïve controls (black bars) and AAV‐injected (gray bars) rats. Error bars indicate *SEM*. *p <* *.05 vs. the value in age‐matched controls at the corresponding time point (Student's *t* test). ^**+**^ *p *<* *.05 vs. the value at the earlier time point in the respective treatment group (age‐matched controls or AAV‐injected rats) (one‐way ANOVA). ^**#**^ *p *<* *.05 vs. the value at the 3‐month time point in the respective treatment group (one‐way ANOVA)

In a final set of analyses, potential pathological changes associated with hα‐synuclein spreading were evaluated in the central amygdala. Following delineation criteria illustrated in Figure S3, the number of Nissl‐stained neurons as well as IBA1‐ and GFAP‐positive cells was counted in the left central amygdala and compared in naïve vs. AAV‐injected rats at different ages and different time points post‐treatment. No significant changes were found when neuronal and astrocyte counts were carried out in tissue specimens from naïve animals of different ages (Figure 5a,c). Similarly, the number of Nissl‐stained and GFAP‐immunoreactive cells remained unaffected in the central amygdala of AAV‐injected rats as compared to age‐matched control animals (Figure 5a,c). Quite in contrast, both aging and AAV treatment had an effect on microglia. Total microglial counts were increased by 10% in naïve rats at 1 year as compared to younger animals (Figure 5b). They were also significantly enhanced at 6 months (+15%) and 1 year (+25%) post‐AAV injection (Figure 5b,d); IBA1‐positive hyper‐ramified and hypertrophic cells contributed to this elevation at both time points (Figure 5e,f).

## 3 DISCUSSION

Evidence supporting a relationship between enhanced expression, neuron‐to‐neuron transfer, and long‐distance spreading of α‐synuclein bears important pathogenetic implications for human synucleinopathies. Several conditions (including aging, environmental insults, genetic variations of the *SNCA* promoter region, and mutations of the glucocerebrosidase gene) are associated with α‐synuclein elevation, underscoring the possibility that protein spreading may also occur under these conditions and contribute to an increased synucleinopathy risk (Mazzulli et al., 2011; Ulusoy & Di Monte, 2013). The present study elucidates important features of overexpression‐induced α‐synuclein transmission and, in particular, reveals long‐lasting effects that could be relevant to disease development and progression in the aging brain. First, data indicate that progressive spreading of α‐synuclein is strictly dependent upon its sustained overexpression within donor neurons. Under the experimental conditions used for this work, increased content of hα‐synuclein within neurons in the medulla oblongata caused its initial brain spreading; on the other hand, a subsequent demise of these donor neurons represented a limiting factor, leading to cessation of caudo‐rostral protein transmission. A second important finding of this study is that, upon death of the donor cells, the number of recipient axons accumulating hα‐synuclein not only did not further increase but actually receded. At least two mechanisms, not mutually exclusive, could account for this long‐term effect. The number of hα‐synuclein‐loaded fibers may decrease as a result of clearance of the exogenous protein, and/or persistent protein load could induce severe axonal injury and, ultimately, death of the recipient neurons. Either of these possibilities is supported by the observation that the mean volume of axonal varicosities, a marker of α‐synuclein burden, declined over time in the pons of AAV‐injected rats. Progressive protein clearance would result in smaller‐sized swellings; alternatively or at the same time, a reduction in mean volume of axonal varicosities could arise from a preferential loss of neurons with greater α‐synuclein burden and larger swellings.

To determine whether protein spreading and consequent axonal hα‐synuclein burden ultimately led to frank neurodegeneration, detailed pathological analyses were carried out and compared in two brain regions, the locus coeruleus and central amygdala. Several reasons justify the choice of these regions. They are both significantly affected by α‐synuclein pathology in the brain of Parkinson's disease patients. According to the Braak staging of disease progression, pathological α‐synuclein lesions appear relatively early in the locus coeruleus (stage 2) and then reach the amygdala at later stages (stages 3 and 4); among the amygdalar nuclei, the central subnucleus represents a primary site of pathological α‐synuclein accumulation (Braak, Rüb, Gai & Del Tredici, 2003 and Braak et al., 2003). Pathological features of both the locus coeruleus and central amygdala in Parkinson's disease brain also include neuronal cell loss, underscoring the relevance of these two regions for studies on the relationship between α‐synuclein pathology and neurodegeneration (German et al., 1992; Harding, Stimson, Henderson & Halliday, 2002). The locus coeruleus and central amygdala densely project to the DMnX and, for this reason, may represent preferential sites of caudo‐rostral α‐synuclein spreading in Parkinson's disease as well as in our present model of overexpression‐induced protein transmission (Braak, et al., 2003). In this model, hα‐synuclein burden in brain regions affected by protein spreading is inversely correlated with their distance from the medulla oblongata and, indeed, the number of hα‐synuclein‐loaded axons and the extent of axonal protein accumulation are much greater in the locus coeruleus (pons) than in the amygdala (forebrain) (Ulusoy et al., 2013). This difference allowed us to assess whether potential long‐term tissue injury was dependent upon severity of the initial α‐synuclein burden.

Findings of this study provide first experimental evidence linking overexpression‐induced α‐synuclein spreading to neurodegeneration. A buildup of hα‐synuclein into axonal projections stemming from the locus coeruleus caused a “dying back” neurodegenerative process and resulted in a progressive loss of catecholaminergic cells in this pontine nucleus. Neurodegeneration did not involve formation of fibrillar or hyperphosphorylated α‐synuclein, consistent with a pathological/toxic role of accumulation of monomeric and oligomeric forms of the protein (Roberts, Wade‐Martins & Alegre‐Abarrategui, 2015; Rochenstain et al., 2014). The sequence of pathological events leading from protein spreading to neurodegeneration is also noteworthy. Neuronal death proceeded after cessation of spreading (at 6 months and 1 year), supporting the concept that even temporary increases in α‐synuclein expression could have sustained consequences in brain regions distant from the site of overexpression but anatomically connected to it.

A marked gliosis characterized the locus coeruleus of AAV‐injected rats. Counts of IBA1‐positive and GFAP‐immunoreactive cells were both significantly enhanced after overexpression‐induced spreading. The time courses of these microglial and astrocytic changes revealed clear differences, however. In particular, a pronounced increase in total microglial number as well as in counts of hypertrophic and amoeboid microglia was already evident at 3 months and was maintained at later time points; marked astrocyte elevations were instead seen only at 6 months and 1 year post‐treatment. Microglial activation at 3 months paralleled and could be a consequence of the initial degeneration of catecholaminergic neurons. It is also possible, however, that these glial changes may be initiated or accentuated by interactions between α‐synuclein and microglia. Earlier work has shown that, once released from neurons, oligomeric α‐synuclein is capable of inducing microglial activation by acting as a ligand of the Toll‐like receptor 2 (TLR2) (Kim et al., 2013). Our results show an elevated number of hα‐synuclein‐loaded axons at 3 months and also reveal that oligomeric α‐synuclein is formed and accumulated within these recipient neurons. Thus, mechanisms leading to an early inflammatory response in the locus coeruleus may include a release of oligomeric α‐synuclein from damaged axons and ensuing TLR2‐mediated microglial activation. The increase in counts of GFAP‐labeled astrocytes displayed a delayed pattern as compared to microglial activation and peaked later than the acute onset of neurodegeneration. It appears to reflect therefore postinjury processes and/or to be part of a more chronic reaction to ongoing degenerative lesions at 6 months and 1 year post‐treatment. Micro‐ and astrogliosis triggered by protein transmission in this model was not accompanied by overt evidence of hα‐synuclein accumulation within these glial cells. This negative observation should be interpreted with a degree of caution, however. Further studies are warranted to rule out the possibility, for example, that a more discrete protein transfer might have been overlooked due to limitations of the detection methods used in our present investigation.

Findings in the locus coeruleus were compared to results of parallel analyses in the central amygdala where, as discussed above, the extent of hα‐synuclein spreading was significantly less pronounced. No evidence of neurodegeneration was found in the central amygdala, thus suggesting that a less severe hα‐synuclein burden is compatible with clearance of the overloaded protein and neuronal survival. Less severe forebrain injury is also likely to explain the lack of astrocytic reaction in the central amygdala of AAV‐injected rats even at late time points post‐treatment. Quite in contrast, an intriguing long‐term effect of hα‐synuclein spreading in the central amygdala was an increase in counts of IBA1‐positive cells at 6 months and 1 year. Taken together, data indicate that astrogliosis is more strictly associated with neurodegenerative changes while activation of microglia can still occur in the absence of severe tissue injury; data are also consistent with the possibility that milder axonal pathology may facilitate the release of hα‐synuclein into the extracellular space and its consequent binding to microglial TLR2. As compared to findings in the locus coeruleus where neuronal cell loss was associated with higher counts of hypertrophic and amoeboid microglia, changes in the central amygdala were accounted for by increases in hyper‐ramified and hypertrophic cells. In line with earlier results (Sanchez‐Guajardo, Febbraro, Kirik & Romero‐Ramos, 2010), these observations suggest that microglial morphology is in part dependent upon the severity of α‐synuclein‐induced pathology; in particular, a marked elevation of IBA1‐positive cells with amoeboid phenotype appears to be a reflection of pronounced tissue injury leading to neurodegeneration.

In summary, results of this study have elucidated a number of important factors and mechanisms affecting overexpression‐induced α‐synuclein spreading and pathology. They include the survival/demise of donor neurons, the extent of protein burden within recipient cells, and the response of brain tissue to α‐synuclein accumulation/release. A potential addition to this list is suggested by our findings in untreated naïve animals. Neuronal counts were similar among naïve rats of different ages in either the locus coeruleus or central amygdala. Age‐related and region‐specific differences were seen, however, in glial counts. In the locus coeruleus, the number of microglia and astrocytes was progressively higher at 6 months and 1 year, whereas, in the central amygdala, a slight but statistically significant elevation of microglia was detected only at 1 year. The pathophysiological relevance of glial changes in the aging brain and their involvement in the pathogenesis of human synucleinopathies are far from being fully understood (Askew et al., 2017; Cotrina & Nedergaard, 2002; Hefendehl et al., 2014). It is reasonable to speculate, however, that age‐ and region‐dependent increases in astrocyte and microglial counts could play a role in modulating long‐term α‐synuclein pathology and may contribute to selective vulnerability to α‐synuclein spreading.

## 4 EXPERIMENTAL PROCEDURES

A detailed description of the experimental procedures can be found in the online supporting information (Appendix S1).

### 4.1 Animals and surgical procedures

Experimental protocols/procedures were approved by the ethical committee of the State Agency for Nature, Environment and Consumer Protection in North Rhine Westphalia. Some of the animals received intravagal injections of recombinant AAVs (serotype 2 genome and serotype 6 capsids) for transgene expression of hα‐synuclein. The surgical procedure for vagal AAV injection has been previously described (Ulusoy et al., 2013).

### 4.2 Tissue preparation and histology

Animals were killed under pentobarbital anesthesia and perfused through the ascending aorta with paraformaldehyde. Brains were removed, immersion‐fixed in paraformaldehyde, and cryopreserved. Coronal sections (40 μm) throughout the brain were cut and stored. Immunohistochemistry was performed on free‐floating sections. The following primary antibodies were used: monoclonal mouse anti‐hα‐synuclein clone syn211 (Merck Millipore, Darmstadt, Germany; 1:10,000), polyclonal rabbit anti‐α‐synuclein (AB5038P, Merck Millipore; 1:750), monoclonal mouse antibody recognizing both α‐synuclein fibrils and oligomers (Syn‐O2, courtesy of Dr. Omar El‐Agnaf; 1:12,000), monoclonal mouse antibody recognizing mature α‐synuclein fibrils (Syn‐F1, courtesy of Dr. Omar El‐Agnaf; 1:10,000), monoclonal rabbit anti‐phospho‐Ser129 α‐synuclein (clone EP15361Y, Abcam, Cambridge, UK; 1:10,000), polyclonal rabbit anti‐GFAP (DAKO**,** Waldbronn, Germany; 1:500), polyclonal rabbit anti‐IBA1 (WAKO, Neuss, Germany; 1:500), polyclonal guinea pig anti‐IBA1 (Synaptic Systems, Goettingen, Germany; 1:500), and polyclonal rabbit antityrosine hydroxylase (Merck Millipore; 1:10,000). All histological quantifications were performed by investigators blinded to the experimental groups. Unbiased stereology using the optical fractionator probe was carried out to estimate cell numbers (neurons, microglia, and/or astrocytes). The number of axons immunoreactive for hα‐synuclein and the volume of hα‐synuclein‐positive axonal varicosities were quantified as previously described (Ulusoy et al., 2013).

### 4.3 Statistical analysis

Statistical analyses were performed with prism software (version 7.0a; GraphPad Software, La Jolla, CA, USA). For normally distributed data, means between two groups were compared with two‐tailed Student's *t* test, and comparisons between multiple groups were carried out with one‐way ANOVA followed by Tukey post hoc test. For non‐normally distributed data, Kruskal–Wallis test was applied. Statistical significance was set at *p* < .05. The number of animals used for each experiment/analysis is indicated in Table S1.

## ACKNOWLEDGMENTS

We thank Sarah A. Jewell for her comments on the manuscript; Omar El‐Agnaf for kindly providing conformation‐specific antibodies; Bettina Winzen‐Reichert, Franziska Hesse, Laura Jakobi for assistance with the experiments; and Ireen Koenig (DZNE Light Microscopy Facility) for assistance with microscopy.

## CONFLICT OF INTEREST

The authors declare that they have no competing interests.

## AUTHOR CONTRIBUTIONS

RR designed experiments and carried out histological analyses, neuronal counts in the DMnX, and volumetric measurements of axonal varicosities; AU performed surgeries and carried out stereological cell counting (neurons and glia) in the central amygdala and axonal counts; HA performed stereological cell counting in the locus coeruleus; DADM designed experiments, analyzed data, and wrote the manuscript with input from the other authors.
