# Supplementary material for: Long‐lasting pathological consequences of overexpression‐induced α‐synuclein spreading in the rat brain
Source: Aging Cell. 2018 Jan 30;17(2):e12727. doi: 10.1111/acel.12727 (PMC5847868; doi:10.1111/acel.12727)
Supplement: Supplementary file 1 [file ACEL-17-e12727-s001.pdf]

“Long-lasting pathological consequences of overexpression-induced α-synuclein spreading in the rat brain”

Raffaella Rusconi, Ayse Ulusoy, Helia Aboutalebi and Donato A. Di Monte

Supplementary figures and supplementary table

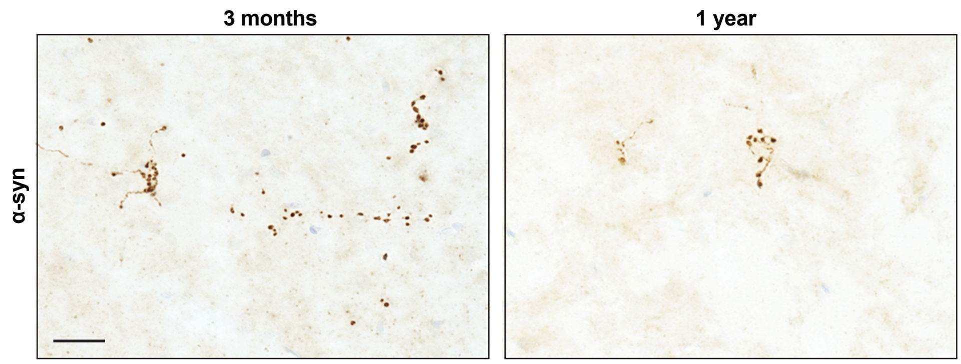

Fig. S1 Axonal pathology caused by hα-synuclein spreading. Pontine sections were stained with AB5038P, an antibody recognizing both human and rat α-synuclein. Representative images show α-synuclein-immunoreactive axons in the left (ipsilateral to the treatment side) pons of rats sacrificed at 3 months or 1 year after vagal AAV injections. Scale bar = 20 µm.

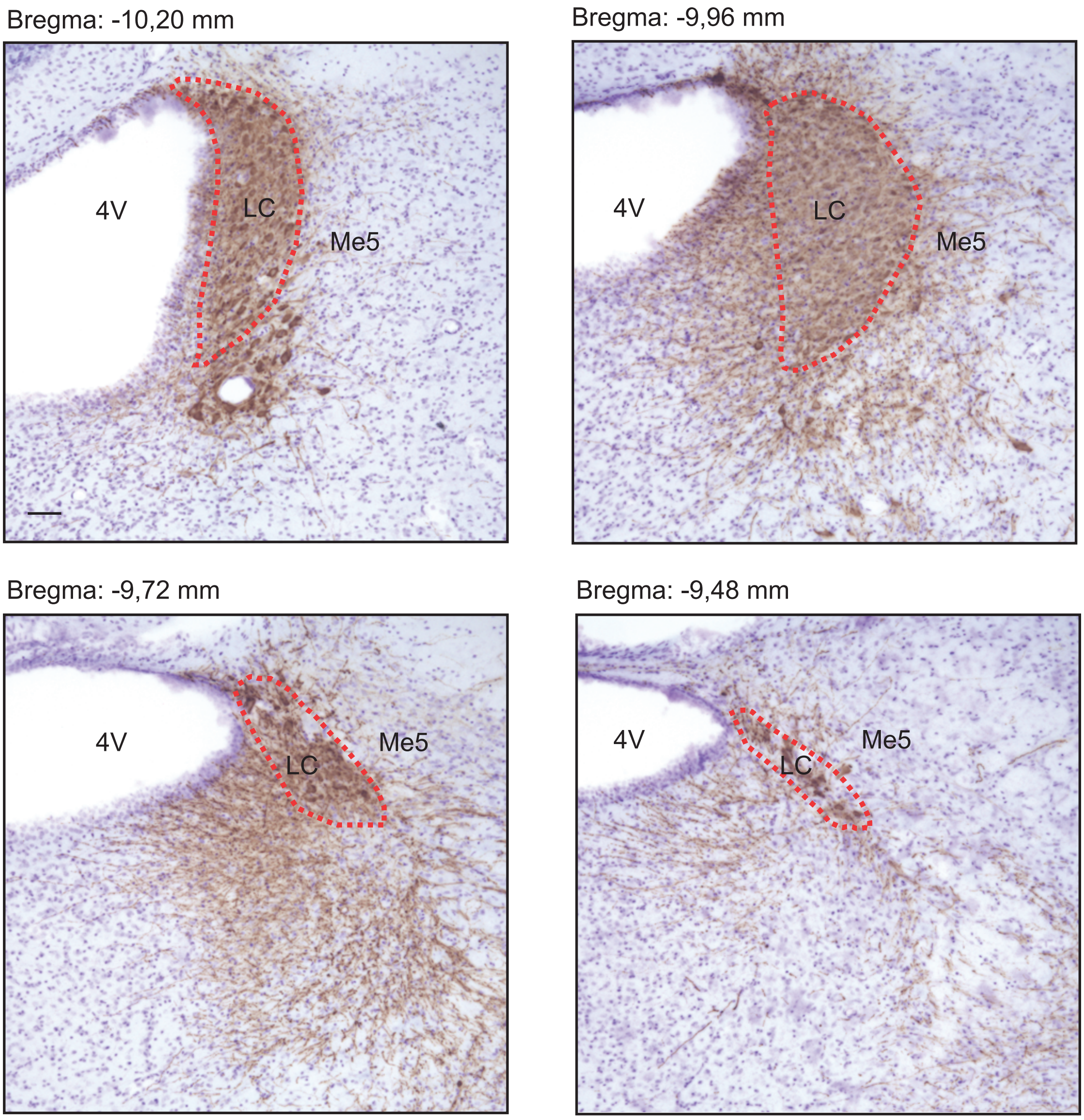

Fig. S2 Delineation of the counting area for stereological analysis in the locus coeruleus. The entire locus coeruleus was encompassed within four coronal sections (40-µm-thick) of the rat pons. Images show each of these sections at the corresponding caudo-rostral Bregma coordinate (-10.20 to -9.48 mm). The locus coeruleus (LC) is delineated by dashed red lines. The mesencephalic trigeminal nucleus (Me5) and 4th ventricle (4V) are also marked. Scale bar = 50 µm.

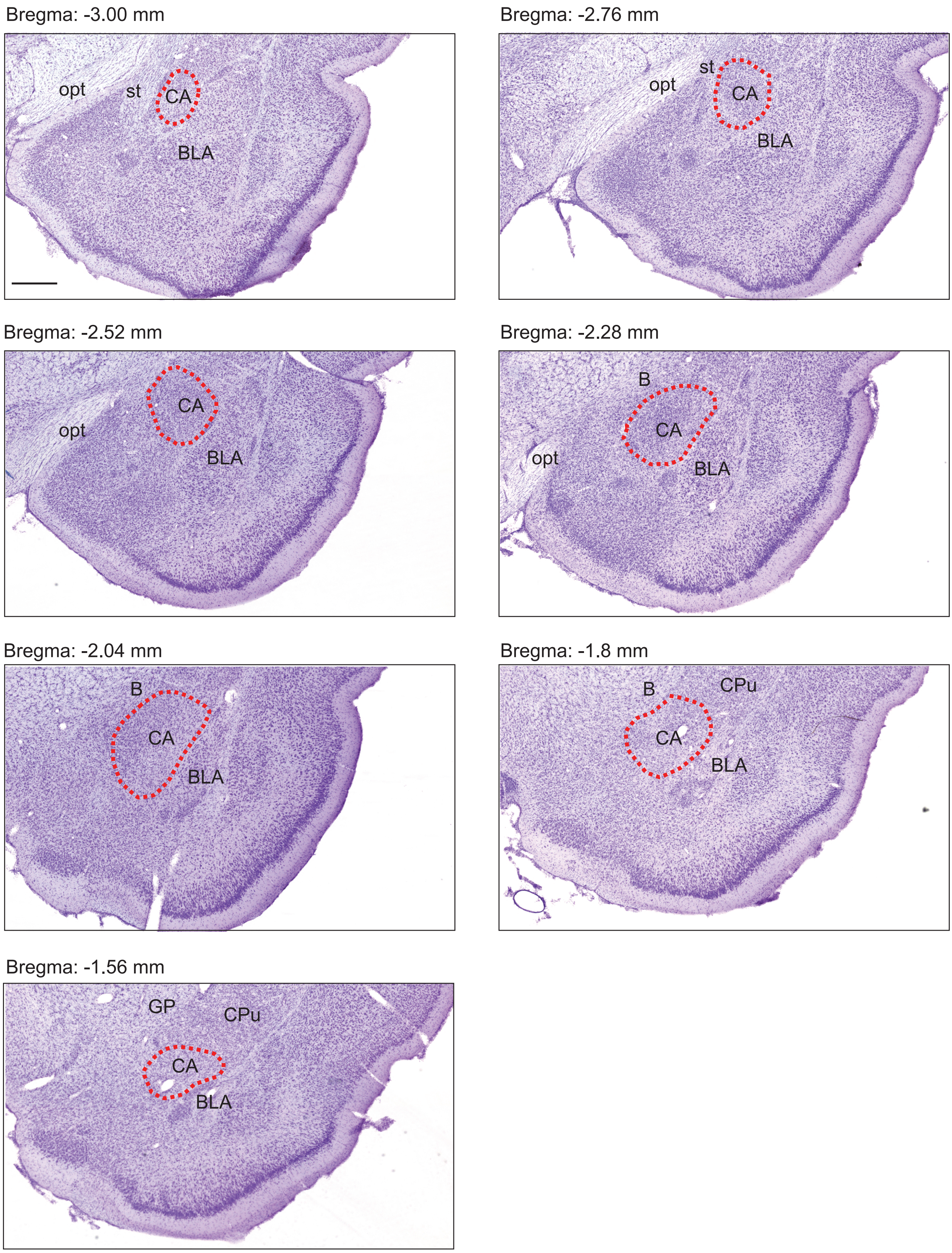

Fig. S3 Delineation of the counting area for stereological analysis in the central amygdala. The entire central amygdala was encompassed within seven coronal sections (40-µm-thick) of the medial temporal lobe. Images show each of these sections at the corresponding caudo-rostral Bregma coordinate (-3.00 to -1.56 mm). The central amygdala (CA) is delineated by dashed red lines, and the following structures/areas are marked: nucleus basalis of Meynert (B); basolateral amygdaloid nucleus (BLA); caudate putamen (CPu); globus pallidus (GP); optic tract (opt); stria terminalis (st). Scale bar = 500 µm.

FIGURE 1

	Fig 1C	Fig 1D	
	ipsi[1]	contra[2]	ipsi
6 weeks	5	6	6
3 months	5	7	8
6 months	6	8	8
1 year	5	5	6

FIGURE 2

	Fig 2A	Fig 2B	Fig 2D
	ipsi	ipsi	ipsi
6 weeks	4	4	
3 months	8	4	4
6 months	12	4	5
1 year	12	4	4

FIGURE 3

	Fig 3D		Fig 3E		Fig 3F	
	naïve[3]	AAV[1]	naïve	AAV	naïve	AAV
6 weeks	4	4	3	4	4	3
3 months	6	5	6	5	6	4
6 months	10	5	6	3	6	4
1 year	8	6	6	5	6	5

FIGURE 4

	Fig 4B	
	naïve	AAV
6 weeks	3	4
3 months	6	5
6 months	6	3
1 year	6	5

FIGURE 5

	Fig 5A		Fig 5B/E/F		Fig 5C	
	naïve	AAV	naïve	AAV	naïve	AAV
6 weeks	3	3	4	3	4	3
3 months	3	4	3	4	3	4
6 months	3	3	4	3	5	4
1 year	4	4	5	4	5	6

Table S1 Number of rats used for the experiments illustrated in Figs. 1-5.

[1]ipsi/AAV = rats received AAV injections, and analyses were made on the left side of the brain, ipsilateral to the injections.

[2]contra = rats received AAV injections, and analyses were made on the right side of the brain, contralateral to the injections.

[3]naïve = analyses were made in control, non-injected rats
