# Supplementary material for: Long‐lasting pathological consequences of overexpression‐induced α‐synuclein spreading in the rat brain
Source: Aging Cell. 2018 Jan 30;17(2):e12727. doi: 10.1111/acel.12727 (PMC5847868; doi:10.1111/acel.12727)
Supplement: Supplementary file 2 [file ACEL-17-e12727-s002.pdf]

“Long-lasting pathological consequences of overexpression-induced α-synuclein spreading in the rat brain”

Raffaella Rusconi, Ayse Ulusoy, Helia Aboutalebi and Donato A. Di Monte

Detailed experimental procedures

Detailed experimental procedures

Vectors

Recombinant AAVs (serotype 2 genome and serotype 6 capsid) were used for transgene expression of hα-synuclein. Gene expression was under the control of the human Synapsin1 promoter and was enhanced using a woodchuck hepatitis virus post-transcriptional element and a polyadenylation signal sequence (Loeb *et al.*, 1999). AAV production and titration were performed by Vector Biolabs (Malvern, PA, USA). Stock preparations were diluted to the injection titer of 1.0 x 10^{13} genome copies/ml.

Animals

Young (3-month-old) adult female Sprague Dawley rats (200-250 g) were purchased from Charles River (Sulzfeld, Germany). Some of these animals received intravagal AAV injections (see below) and were killed at different time points (6 weeks, 3 months, 6 months or 1 year) post treatment. Naïve, untreated rats were used as controls. The age of these animals was matched to the age of treated rats at the different experimental end points. For example, age-matched controls for the experimental group of AAV-injected rats killed at 1 year were 15-month-old. All animals were housed under a 12h light/12h dark cycle with *ad libitum* access to food and water.

Surgical procedures

Experimental protocols/procedures were approved by the ethical committee of the State Agency for Nature, Environment and Consumer Protection in North Rhine Westphalia. The surgical procedure for vagal AAV injection has been previously described (Ulusoy *et al.*, 2013). Following anesthesia with 2% isoflurane mixed with O_2 and N_2O, a 2 cm incision was made along the midline of the rat neck. The left vagus nerve was isolated from the surrounding tissue, and viral vector solution (2 μl) was injected at a flow rate of 0.5 μl/min using a glass capillary (tip diameter = 60 μm) fitted to a 5 μl Hamilton syringe. The capillary was left in place for 2 min after injection.

Tissue preparation

Animals were killed under pentobarbital anesthesia and perfused through the ascending aorta with saline, followed by ice-cold 4% (w/v) paraformaldehyde. Brains were removed, immersion-fixed in 4% paraformaldehyde for 24 h and cryopreserved in 25% (w/v) sucrose solution. Coronal sections (40 μm) throughout the brain were cut using a freezing microtome and stored at -20°C in phosphate buffer (pH 7.4) containing 30% glycerol and 30% ethylene glycol.

Histology

Immunohistochemistry was performed on free-floating sections. Samples were rinsed in Tris buffer saline (TBS, pH 7.6), quenched for 1 hour in TBS containing 3% H_2O_2/10% methanol, and blocked for 1 hour in 5% normal serum in TBS containing 0.25% Triton-X-100 (TBS-T). Sections were incubated overnight at room temperature with the primary antibody in TBS-T solution containing 1% BSA. The following primary antibodies were used: monoclonal mouse anti-hα-synuclein clone syn211 (Merck Millipore, Darmstadt, Germany; 1:10,000), polyclonal rabbit anti-α-synuclein (AB5038P, Merck Millipore; 1:750), monoclonal mouse antibody recognizing both α-synuclein fibrils and oligomers (Syn-O2, courtesy of Dr. Omar El-Agnaf; 1:12,000), monoclonal mouse antibody recognizing mature α-synuclein fibrils (Syn-F1, courtesy of Dr. Omar El-Agnaf; 1:10,000), monoclonal rabbit anti-phospho-Ser129 α-synuclein (clone EP15361Y, Abcam, Cambridge, UK; 1:10,000), polyclonal rabbit anti-GFAP (DAKO, Waldbronn, Germany; 1:500), polyclonal rabbit anti-IBA1 (WAKO, Neuss, Germany; 1:500), polyclonal guinea pig anti-IBA1 (Synaptic Systems, Goettingen, Germany; 1:500) and polyclonal rabbit anti-tyrosine hydroxylase (Merck Millipore; 1:10,000). They were then incubated with a species-specific secondary antibody (Vector Laboratories, Burlingame, CA, USA; 1:200) for 1 hour at room temperature. Following 1 hour treatment with streptavidin-horseradish peroxidase complex (ABC Elite kit, Vector Laboratories), the staining was visualized using 3,3′-diaminobenzidine kit (Vector Laboratories). Sections were finally mounted on coated slides, dried, counterstained (if necessary) with cresyl violet (FD Neurotechnologies, Columbia, MD, USA) and cover-slipped with Depex (Sigma-Aldrich, Munich, Germany). To detect α-synuclein aggregates with Syn-O2 or Syn-F1, a few modifications were made in the staining procedure. Sections were

quenched for 30 minutes in Bloxall blocking solution (Vector Laboratories). An antigen retrieval step, which was shown to improve staining of immunohistochemical brain sections (Vaikath *et al.*, 2015), was also performed before blocking in normal serum: sections were incubated for 5 min at room temperature with 1.5 mg/L proteinase K (Sigma-Aldrich).

Histological quantifications

All quantifications were performed by investigators blinded to the experimental groups. Unbiased stereology using the optical fractionator probe was carried out to estimate cell numbers (neurons, microglia and/or astrocytes) in three different brain regions: DMnX, locus coeruleus and central amygdala. Tissue sections were visualized and analyzed on either a Nikon Eclipse 90i (Nikon, Düsseldorf, Germany) or an Olympus BX51 WI (Olympus, Hamburg, Germany) microscope equipped with Stereo Investigator software version 10 (MBF Biosciences, Williston, VT, USA). Delineation of the DMnX was performed with a 10x objective as previously described (Ulusoy *et al.*, 2015). The locus coeruleus and central amygdala were delineated using a 10x- and 4x-magnifying objective, respectively. Delineation criteria are illustrated in Fig. S2 and S3. Coefficient of error was calculated according to Gundersen and Jensen (Gundersen and Jensen, 1987) and values <0.10 were accepted.

The number of axons immunoreactive for hα-synuclein or total (human plus rodent) α-synuclein was quantified in sections at predefined Bregma coordinates (Paxinos & Watson, 2009): -9.60 mm (pons), -7.80 mm (caudal midbrain), - 6.00 mm (rostral midbrain) and - 2.40 (forebrain). Immunoreactive fibers were counted using an Axioscope microscope (Carl Zeiss, Göttingen, Germany) under a 40x Plan-Apo objective. Stack images were collected on an Olympus BX51 WI microscope at 0.5 µm intervals with a 60x oil immersion objective (Numerical aperture = 1.4). Single images were then generated using deep focus post-processing.

The volume of hα-synuclein-positive axonal varicosities was estimated in pontine sections after delineation of an area encompassing the locus coeruleus, the parabrachial nucleus and the subcoeruleus nucleus. Swellings were visualized under a 60x oil immersion objective. The volume of every axonal swelling present in the delineated area

was measured using the isotropic nucleator probe of the Stereo Investigator software version 10 (Ulusoy *et al.*, 2013). Varicosities with a volume greater than 0.5 μm^3 were included in the analysis. Mean volumes data were expressed as geometric mean ± 95% confidence interval.

Statistical analysis

Statistical analyses were performed with Prism software (version 7.0a; GraphPad Software, La Jolla, CA, USA). For normally distributed data, means between two groups were compared with two-tailed Student's t-test, and comparisons between multiple groups were carried out with one-way ANOVA followed by Tukey *post hoc* test. For non-normally distributed data, Kruskal-Wallis test was applied. Statistical significance was set at $P < 0.05$. The number of animals used for each experiment/analysis is indicated in Table S1 (supporting information).

Supplemental references

Gundersen, H.J., & Jensen, E.B. (1987). The efficiency of systematic sampling in stereology and its prediction. *Journal of Microscopy, 147*(Pt 3), 229-263

Loeb, J.E., Cordier, W.S., Harris, M.E., Weitzman, M.D., & Hope, T.J. (1999). Enhanced expression of transgenes from adeno-associated virus vectors with the woodchuck hepatitis virus posttranslational regulatory element: implication for gene therapy. *Human Gene Therapy, 10*(14), 2295-2305

Paxinos, G., & Watson, C. (2009). *The rat brain in stereotaxic coordinates*. London, UK: Academic Press.

Ulusoy, A., Musgrove, R.E., Rusconi, R., Klinkenberg, M., Helwig, M., Schneider, A., & Di Monte, D.A. (2015). Neuron-to-neuron α-synuclein propagation in vivo is independent of neuronal injury. *Acta Neuropathologica Communications, 24*, 3-13. https://doi: 10.1186/s40478-015-0198-y
